# Improvement of brain perfusion in patients with chronic brain ischemia at epidural spinal cord electrical stimulation

**DOI:** 10.3389/fsurg.2022.1026079

**Published:** 2022-09-23

**Authors:** Shu Zhao, Galina Sufianova, Andrey Shapkin, Andrey Mashkin, Svetlana Meshcheryakova, Dayong Han

**Affiliations:** ^1^ Emergency Medicine, First Affiliated Hospital of Harbin Medical University, Harbin, China; ^2^ Department of Pharmacology, Tyumen State Medical University, Tyumen, Russia; ^3^ Department of Functional Neurosurgery, Federal Center of Neurosurgery, Tyumen, Russia; ^4^ Educational and Scientific Institute of Neurosurgery, Peoples' Friendship University of Russia (RUDN University), Moscow, Russian; ^5^ Department of General Chemistry, Bashkir State Medical University, Ufa, Russia; ^6^ Department of Neurosurgery, First Affiliated Hospital of Harbin Medical University, Harbin, China

**Keywords:** cerebral blood flow, electrical spinal cord stimulation, chronic cerebral ischemia, cerebrovascular diseases, CT perfusion

## Abstract

**Objective:**

Increasing life expectancy and aging of the population is accompanied by a steady increase in the number of elderly patients with chronic cerebral ischemia and age-related cognitive impairment associated with cerebral hypoperfusion and microangiopathy. The aim of this study was to identify long-term changes in cerebral blood flow (CBF) in patients with chronic cerebral ischemia at the epidural electrical stimulation of the spinal cord (SCS).

**Materials and methods:**

Changes in cerebral blood flow were studied according to CT perfusion in 59 patients (aged 55–78 years) with vertebrogenic pain syndromes and chronic cerebral ischemia during epidural electrical stimulation of the spinal cord at the cervical (C3–C5) and lower thoracic (Th9–Th10) levels.

**Results:**

In all patients, on the 5th day of trial SCS, an increase in cerebral blood flow by from 58.6 ± 1.13 ml/100 ml/min to 64.8 ± 1.21 ml/100 ml/min (*p* < 0.01) with stimulation at the Th9-Th10 level and from 58.8 ± 1.12 ml/100 ml/min to 68.2 ± 1.42 ml/100 ml/min (*p* < 0, 01) with stimulation at the C3-C5 level. These changes in brain perfusion were preserved during the follow-up examination 1 year after the implantation of chronic SCS system. The greatest increase in CBF was registered in the frontotemporal regions, subcortical structures and white matter of the brain. Changes in cerebral perfusion did not correlate with the degree of reduction in the severity of the accompanying pain syndrome. The change in CBF in the control group (32 patients) in all periods was not statistically significant.

**Conclusion:**

Our results show that SCS is accompanied by a persistent improvement in brain perfusion, which may be potentially useful for developing methods for reducing age-related vascular disorders in the elderly.

## Introduction

The increase in life expectancy and aging of the population is accompanied by a steady increase in the number of elderly patients with chronic cerebral ischemia and age-related cognitive impairment associated with cerebral hypoperfusion and microangiopathy (1–5). The high social significance and prevalence of these disorders determines the relevance of finding new ways to correct these conditions, however, existing pharmacological methods that improve the functional state of the central nervous system (CNS) do not always meet the expectations of both doctors and patients (3, 4). In recent years, various methods of neuromodulation based on epidural electrical stimulation of the spinal cord have been proposed in clinical practice for the correction of peripheral hemodynamic disorders in patients suffering from refractory angina pectoris or peripheral vasculopathy (6–11). Also, in many experimental and clinical studies, a positive effect of electrical stimulation of the spinal cord on the hemodynamic and functional parameters of the brain was noted in certain pathological conditions associated with dysfunction of the vascular bed, such as ischemia, subarachnoid hemorrhage, head trauma and brain tumors (10, 12–16). Most of the conducted clinical and experimental studies devoted to the study of the short-term effect of the upper (at the level of C2–C3 of the cervical spinal cord) electrical stimulation of the spinal cord were performed, as a rule, in acute and severe circulatory disorders, accompanied by critical neurological disorders and the development of a vegetative state, such as stroke, vasospasm, or traumatic brain injury (TBI) (17–21). At the same time, studies of the effect of prolonged electrical stimulation in moderate chronic perfusion disorders of the brain have not been conducted. The purpose of this study was to study long-term changes in local cerebral blood flow in patients with chronic cerebral ischemia on the background of epidural electrical stimulation of the spinal cord.

## Materials and methods

### Patients

A prospective longitudinal study was conducted with the participation of 91 patients aged 55–78 years (mean age 66.9 ± 0.7 years, 52 men, 39 women) hospitalized in the 5th neurosurgical department (functional neurosurgery) of the Federal Center for Neurosurgery, Tyumen, Russia, in the period from 2015 to 2021. The study was approved by the local ethical committee (No. 2 dated April 21, 2014). Consent was taken from all patients or their representatives, which is included in the standard form upon admission to a medical institution for the necessary diagnostic and therapeutic measures in a neurosurgical hospital in accordance with the legislation of the Russian Federation. Surgical interventions were performed by the same operating team with minimal surgical invasion.

**Figure 1:**
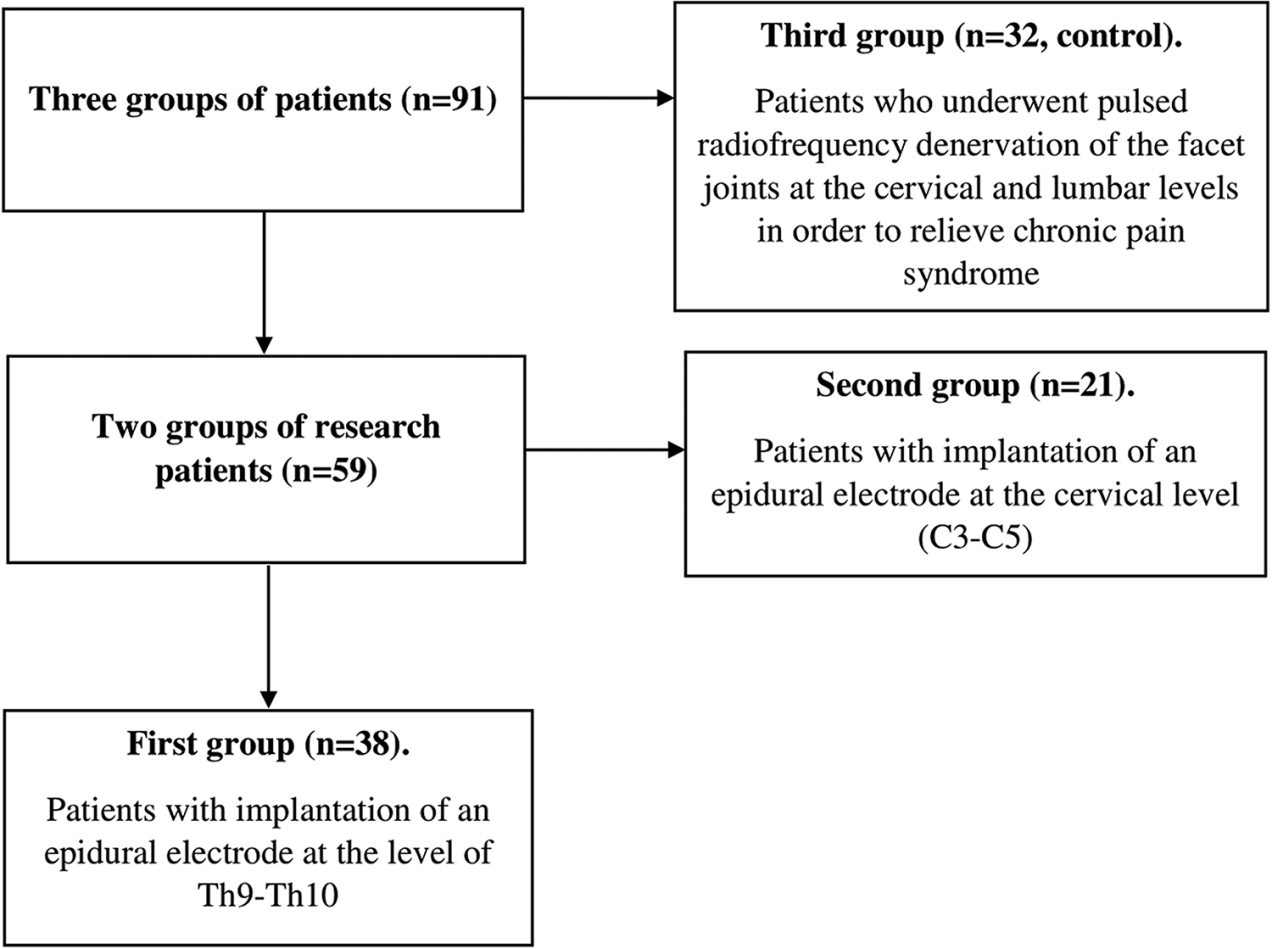
Flow-chart of formation of groups for the study.

In accordance with the purpose of the study, 3 groups of patients were recruited (Figure 1). Patients of all groups were treated for severe pain syndrome of vertebrogenic origin. Groups were formed in accordance with the inclusion and exclusion criteria (Table 1). The first group included 59 patients who underwent temporary epidural electrical stimulation of the spinal cord with subsequent possible implantation of a system of chronic epidural electrical stimulation. Depending on the location of the implantable epidural electrode, this group was divided into 2 main subgroups. The first clinical subgroup (38 patients, mean age 67.3 ± 1.1 years, 22 men, 16 women) included patients with implantation of an epidural electrode at the Th9–Th10 level. In the second clinical subgroup, the epidural electrode was implanted at the cervical level (C3–C5). This group included 21 patients (mean age 66.7 ± 1.7 years, 12 men, 9 women). The control group included 32 patients (18 men and 14 women, mean age 66.4 ± 1.3 years), who underwent pulsed radiofrequency denervation of the facet joints at the cervical and lumbar levels in order to relieve chronic pain. In all groups, various combinations of non-steroidal anti-inflammatory drugs (NSAIDs) and non-narcotic analgesics were used to relieve pain before surgery. In the postoperative period, drug therapy was not carried out. Temporary electrodes were implanted percutaneously under local anesthesia.

**Table 1:** Inclusion and exclusion criteria.

Inclusion criteria	Exclusion criteria
Signs of chronic cerebral ischemia (grade 2–3 according to the Fazekas visual scale), confirmed by the results of MRI tomography of the brain	Excluded were patients who continued to require high doses of NSAIDs, narcotic analgesics, and drugs for the treatment of neuropathic pain in the postoperative period.
Stenosis of the brachiocephalic arteries less than 50%, not requiring surgical treatment or its absence	
No history of acute disorders of cerebral circulation with the development of an organic defect in brain structures	
No indications for “open” surgical interventions on the spine (spinal canal stenosis, disc herniation, etc.)	
Absence of decompensated somatic complications not associated with ischemic brain damage	
Chronic vertebrogenic pain syndrome in the cervical or lumbar spine according to VAS more than 5 and lasting from 6 months to 2–3 years	

MRI, magnetic resonance imaging; VAS, visual analog scale; NSAIDs, non-steroidal anti-inflammatory drugs.

### Technique

The period of test electrical stimulation in patients of the first group, on average, was 7.1 ± 1.2 days. For temporary epidural electrical stimulation, 8-contact trial electrodes (St. Jude Medical Inc., USA) were used. Rechargeable Restore Sensor (Medtronic, USA) and EON Mini (St. Jude Medical Inc., USA) systems were implanted for chronic epidural electrical stimulation. Standard stimulation parameters were set—80 Hz, 500 μs. The stimulation amplitude was selected individually to the level of comfortable paresthesias in the lower or upper extremities, on average from 2.5 to 6 mA (2–5 mV in the case of Medtronic stimulators). Patients were advised to conduct continuous electrical stimulation of the spinal cord for at least 12 h a day. The effectiveness of epidural stimulation of the spinal cord was assessed by reducing the severity of pain and improving the quality of life of patients. To assess the severity of the pain syndrome, a 10-point visual analog pain scale (VAS) was used.

To detect manifestations of microangiopathy in chronic cerebral ischemia, magnetic resonance imaging (MRI) in the T2-WI mode (FLAIR) was used. The study was carried out on a 3 T MR tomograph General Electric Discovery W750. For the purpose of quantitative assessment of the severity of this phenomenon, the Fazekas visual scale (1998) was used: 0—absence of leukoaraiosis; 1—mild leukoaraiosis; 2—moderate confluent leukoaraiosis; 3—severe confluent leukoaraiosis. Changes in cerebral hemodynamics were assessed using the computed tomography (CT) perfusion method on a Canon Aquilion ONE Next Generation tomograph (Canon Medical Systems Europe, Germany). In the first group, the study was carried out with the neurostimulator turned on. Scanning was performed after injection of 40 ml of a non-ionic contrast agent containing 300 mg of iodine per ml (omnipaque), at an injection rate of 8 ml/s; the time of complete infusion with an automatic injector is 5 s. Four seconds after the start of the injection, a 40 s continuous scan was obtained at the selected slice site at 1 s intervals. The results were evaluated both graphically and visually, followed by color mapping of the images. The following parameters were studied: cerebral blood volume (CBV, ml/100 g), cerebral blood flow (CBF, ml/100 g/min) and mean transit time (MTT, s). These parameters were calculated for the cerebral cortex as a whole and separately for the frontal, temporal, parietal, and occipital lobes, as well as the thalamus, basal ganglia, anterior cingulate gyrus, and white matter. Data for the right and left hemispheres, taking into account the symmetry of cerebral blood flow disorders in the examined patients, were averaged. CT perfusion was performed upon admission to the hospital, on the 5th day and at the control examination after 1 year.

### Statistical analysis

Statistical analysis and visualization of the study results were performed using MS Office Excel 2021 and Matlab 2019 (MathWorks, Inc., Natick, MA, USA). To assess the statistical significance of the results obtained, the parametric *t*-student test and the nonparametric *U*-test—Wilcoxon–Mann–Whitney were used. The results are presented as *M* ± *m*, where *M* is the arithmetic mean and *m* is the error of the mean. Differences were considered significant at *p *≤ 0.05.

## Results

### CT perfusion data

**Figure 2:**
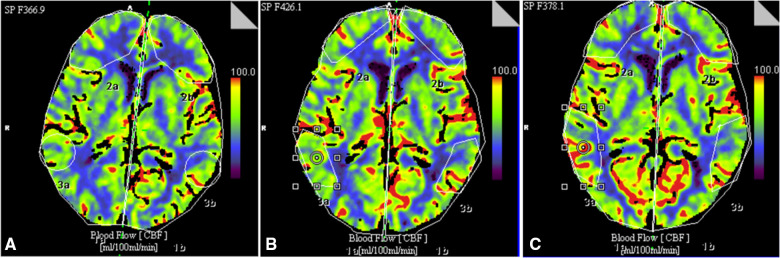
An example of changes in CBF in a 78-year-old patient with chronic cerebral ischemia before implantation of the stimulation system (**A**), on day 5 (**B**) and 1 year (**C**) after implantation of the spinal cord system at the level of Th9–Th10.

All patients of the main clinical group on the background of electrical stimulation subjectively described an improvement in well-being, normalization of sleep and a decrease in the level of anxiety and depression, cognitive functions. According to the visual 10-point analog scale, the intensity of pain syndrome on the background of electrical stimulation decreased in subgroup 1 from 6.6 ± 0.19 to 2.5 ± 0.1 (*p *≤ 0.01) on day 5 and 1.8 ± 0.2 score after 1 year (*p *≤ 0.01). In the second subgroup, the intensity of the pain syndrome decreased from 6.7 ± 0.2 to 2.5 ± 0.2 (*p *≤ 0.01) on day 5 and 1.9 ± 0.2 points after 1 year (*p *≤ 0.01). In the control group, in all patients, the level of pain syndrome decreased in the postoperative period from 6.4 ± 0.2 to 1.6 ± 0.18 (*p *≤ 0.01) on day 5. At the follow-up examination, almost all patients of this group experienced recurrence of pain syndrome, which averaged 4.3 ± 0.3 in the group (*p *≤ 0.01 compared with the initial level). An increase in the intensity of the pain syndrome by more than 3 points was observed in 62.5% of patients. The change in local cerebral blood flow in patients of the control group in all periods was not statistically significant. In all patients of the main group, both with stimulation of the lower thoracic and cervical sections of the spinal cord, an improvement in cerebral circulation was noted according to CT perfusion of the brain (Figure 2).

### Cerebral cortex

**Figure 3:**
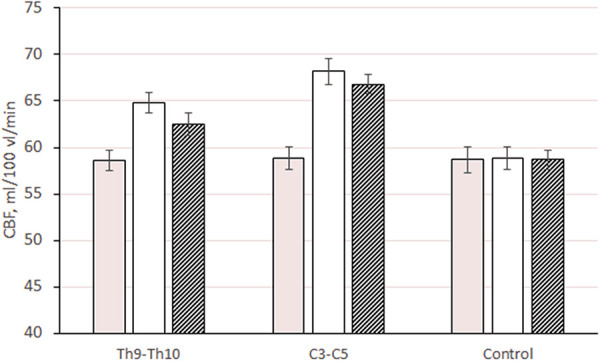
Changes in CBF (ml/100 ml/min) in the cerebral cortex (on average for all departments) in patients with epidural electrode placement at different levels: Th9–Th10, 1 subgroup; C3–C5, 2 subgroups; control, control group.

In patients of the first and second subgroups, in the first 5 days of stimulation, local cerebral blood flow in the cerebral cortex increased by 10.6% and 15.9% of the initial (preoperative level): from 58.6 ± 1.13 to 64.8 ± 1.21 ml/100 ml/min (*p *≤ 0.01 compared to baseline and changes in the control group) and from 58.8 ± 1.12 to 68.2 ± 1.42 ml/100 ml/min (*p *≤ 0.01 versus baseline and change in control group, *p *≤ 0.01 versus subgroup 1) (Figure 3). The increase in cerebral perfusion in patients of the first and second subgroups in the cerebral cortex was higher than the initial preoperative level by 6.6% and 13.6%. The level of cerebral perfusion in patients during this period was, respectively, 62.5 ± 1.39 ml/100 ml/min (*p *≤ 0.01 compared with baseline and changes in the control group) and 66.8 ± 1.22 ml/min. 100 ml/min (*p *≤ 0.01 compared to baseline and changes in the control group, *p *≤ 0.01 compared to 1 subgroup).

### White matter

**Figure 4:**
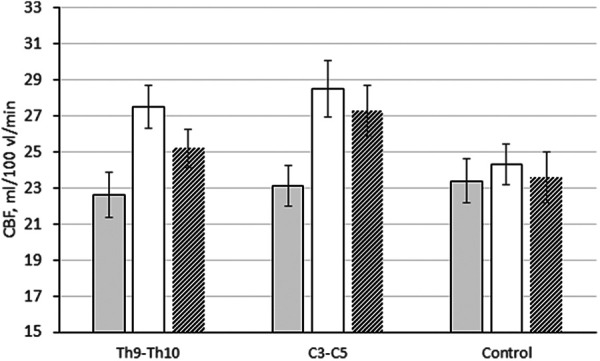
Changes in CBF (ml/100 ml/min) in the white matter of the brain in patients with epidural electrode placement at different levels: Th9–Th10, subgroup 1; C3–C5, subgroup 2; control, control group.

In the white matter, brain perfusion increased by 21.6% and 23.4%, respectively: from 22.6 ± 1.26 to 27.5 ± 1.13 ml/100 ml/min (*p *≤ 0.01 compared with baseline and changes in the control group) and from 23.1 ± 1.19 to 28.5 ± 1.57 ml/100 ml/min (*p *≤ 0.01 in compared with baseline and changes in the control group) (Figure 4). At the control study after 1 year, this trend persisted. Local cerebral blood flow in the white matter remained higher than the initial level by 11.5% (25.2 ± 1.39 ml/100 ml/min, *p *≤ 0.01 compared with the initial level and changes in the control group) and 18.2% (27.3 ± 1.12 ml/100 ml/min (*p *≤ 0.01 compared to baseline and changes in the control group), respectively.

### General data

The maximum increase in cerebral blood flow was observed in the frontal (12%–24%), temporal cortex (11%–18%), cingulate gyrus (15%–17%) and subcortical nuclei (thalamus, basal ganglia—16%–20%). Minimal changes in the parietal and occipital cortex—4%–10% (Table 2). Changes in MTT and CBV in all groups and in individual brain structures were not significant. MTT before surgery and in other periods averaged 8.9 ± 0.18 s in patients of different groups, CBV—3.7 ± 0.15 ml/100 g. Changes in brain perfusion did not correlate with the degree of pain reduction.

**Table 2:** Changes in cerebral blood flow (CBF) (ml/100 ml/min) in different parts of the brain in patients of different groups.

	1 Group (Th9–Th10)	2 Group (С3–С5)	3 Group (control)
**Before stimulation**			
Frontal cortex	56.3 ± 1.08	56.1 ± 1.16	56.8 ± 1.12
Temporal cortex	58.4 ± 1.19	58.8 ± 1.3	59.2 ± 1.26
Parietal cortex	61.5 ± 1.02	62.1 ± 1.25	61.6 ± 1.3
Occipital cortex	61.8 ± 1.17	61.6 ± 1.18	60.3 ± 1.1
Cingulate gyrus	55.6 ± 1.16	56.1 ± 1.06	55.9 ± 1.06
Thalamus	60.3 ± 1.09	62.3 ± 1.22	59.4 ± 1.08
Basal ganglia	55.8 ± 1.02	56.1 ± 1.3	56.1 ± 1.14
Cerebral cortex (all parts)	58.6 ± 1.13	58.8 ± 1.12	58.7 ± 1.17
White matter of the brain	22.6 ± 1.26	23.1 ± 1.19	23.4 ± 1.05
**5 Days**			
Frontal cortex	63.6 ± 1.13*	69.9 ± 1.3*	57.6 ± 1.11
Temporal cortex	65.2 ± 0.98*	69.8 ± 1.31*	58.8 ± 1.21
Parietal cortex	66.2 ± 1.4*	68.3 ± 1.33*	61.3 ± 1.32
Occipital cortex	64.8 ± 1.15*	66.8 ± 1.47*	59.9 ± 1.17
Cingulate gyrus	64.1 ± 1.26*	66.1 ± 1.62*	56.6 ± 1.14
Thalamus	68.9 ± 1.02*	68.3 ± 1.36*	57.2 ± 1.15
Basal ganglia	67.1 ± 0.94*	65.1 ± 1.61*	56.5 ± 1.3
Cerebral cortex (all parts)	64.8 ± 1.21*	68.2 ± 1.42*	58.8 ± 1.05
White matter of the brain	27.5 ± 1.13*	28.5 ± 1.57*	24.3 ± 1.39
**1 Year after surgery**			
Frontal cortex	62.2 ± 1.09*	67.8 ± 1.31*	57.5 ± 1.11
Temporal cortex	63.7 ± 1.38*	68.3 ± 1.07*	58.2 ± 1.21
Parietal cortex	63.5 ± 1.07	66.6 ± 1.2*	62.1 ± 1.32
Occipital cortex	60.9 ± 1.35	66.2 ± 1.29*	60.8 ± 1.17
Cingulate gyrus	62.4 ± 1.34*	65.3 ± 1.27*	55.3 ± 1.14
Thalamus	65.5 ± 1.13b*	68.8 ± 1.18*	55.7 ± 1.15
Базальные ганглии	64.2 ± 1.09*	65.5 ± 1.2*	56.9 ± 1.3
Cerebral cortex (all parts)	62.5 ± 1.39*	66.8 ± 1.22*	58.7 ± 1.05
White matter of the brain	25.2 ± 1.23*	27.3 ± 1.12*	23.6 ± 1.39

* *p* ≤ 0.01 and *p* ≤ 0.05 compared to baseline.

## Discussion

In connection with the development of medicine and improvement in the quality of life in the last 100 years, there has been an increase in the life expectancy of people all over the world, accompanied by a general aging of the population and an increase in the number of “age-related diseases”, including cognitive disorders of various origins (2, 3, 22). From 3% to 20% of people over 65 years of age have severe cognitive impairment in the form of dementia (3, 23). The incidence of milder cognitive impairment in the elderly is even greater, reaching, according to some reports, from 40% to 80% depending on age (24, 25). The leading etiological factors in the development and aggravation of chronic cerebral ischemia are cerebral atherosclerosis, arterial hypertension, and diabetes mellitus (2, 5, 23–25). The increase in the prevalence of cognitive impairments with increasing age of patients indicates their connection with changes in the brain that naturally develop as it ages. These changes include a decrease in the number of neurons (by 0.1%–0.2% for each year after 50 years), their dendrites, synapses, receptors, as well as the loss of glial elements (26). The consequence of this is a decrease in the volume of the brain and its individual parts, a decrease in the level of metabolism and perfusion of the brain. Atrophy primarily affects the frontal lobes (volume decreases by 0.5% per year) and temporal lobes (volume decreases by 0.3% per year), as well as deep regions, which leads to expansion (by 3.2% per year) of the lateral ventricles of the brain (26–28). According to positron emission tomography (PET), the activity of the frontal cortex is the first to decrease with aging (28). Hypoxic changes in the brain, which cause the appearance of neurological disorders and decompensation of a neuropsychic defect in cerebrovascular pathology, are largely determined by the reserve capabilities of central and cerebral hemodynamics (25, 27, 28). Cognitive impairment is often accompanied by emotional and personality changes: irritability, passivity, apathy, symptoms of depression (26). The presence of various concomitant chronic diseases accompanied by pain can exacerbate the picture of cognitive impairment in elderly patients. Existing pharmacological methods for correcting chronic disorders of cerebral perfusion do not always meet the expectations of both doctors and patients or their relatives (29). The presence of side effects of drugs in some cases does not allow their use in patients with concomitant diseases. In addition, most nootropic drugs and neurometabolic stimulants used to improve cognitive status do not have objective evidence of their effectiveness (30). Taking into account the ambiguous action, side and non-selective effects of drugs used in neurology, non-pharmacological approaches to the correction of neuropsychiatric disorders are currently becoming popular. For this purpose, methods of magnetic and electrical stimulation of the central nervous system are widely used. Electrical stimulation of the central and peripheral parts of the nervous system (stimulation of the structures of the autonomic nervous system, epidural electrical stimulation of the spinal cord, electrical stimulation of individual nerves and nerve bundles) has now found wide application and has shown high efficiency in clinical practice for the functional correction of many neurological and neurosurgical diseases, such as Parkinson's disease, essential tremor, epilepsy, spastic and pain syndromes (6, 11, 13, 31). Considering the high frequency of concomitant “age-related” diseases accompanied by chronic pain syndromes, we conducted a study to identify possible positive effects of electrical spinal cord stimulation in elderly patients with chronic cerebral ischemia. We selected a group of patients with verified chronic cerebral ischemia with signs of microangiopathy according to MRI studies (23, 32). In our study, we have shown for the first time the effectiveness of chronic epidural electrical stimulation of the spinal cord in chronic cerebral ischemia. Given that perfusion changes are observed mainly in the frontoparietal cortex, our results may be potentially useful for developing methods for correcting cognitive impairment in patients with various forms of dementia, as well as for reducing age-related vascular disorders in the elderly (15, 27, 28). Mechanisms for improving cerebral blood flow during electrical stimulation of the spinal cord have not been sufficiently studied (17, 18, 21). Most publications discuss the effect of electrical stimulation of the upper cervical spinal cord in the acute period of vascular disorders such as TBI, subarachnoid hemorrhage, stroke, or in patients in a vegetative state (14, 17, 19, 20). It is assumed that the improvement in brain perfusion may be associated with neurodynamic changes and a decrease in the functional activity of the sympathetic nervous system, improved autoregulation of cerebral circulation, as well as the influence of neurohumoral factors and the accumulation of endogenous vasodilator substances in the CNS structures (17–21, 33).

## Conclusion

The “functional” cerebral revascularization observed by us in chronic ischemia in the elderly seems promising as a new direction in functional neurosurgery in cerebrovascular diseases.

## Data Availability

The original contributions presented in the study are included in the article/Supplementary Material, further inquiries can be directed to the corresponding author/s.
